# Polymerizing Pyrrole Coated Poly (l-lactic acid-co-ε-caprolactone) (PLCL) Conductive Nanofibrous Conduit Combined with Electric Stimulation for Long-Range Peripheral Nerve Regeneration

**DOI:** 10.3389/fnmol.2016.00117

**Published:** 2016-11-08

**Authors:** Jialin Song, Binbin Sun, Shen Liu, Wei Chen, Yuanzheng Zhang, Chunyang Wang, Xiumei Mo, Junyi Che, Yuanming Ouyang, Weien Yuan, Cunyi Fan

**Affiliations:** ^1^Shanghai Jiao Tong University Affiliated Sixth People’s HospitalShanghai, China; ^2^Shanghai Sixth People’s Hospital East Campus, Shanghai University of Medicine and HealthShanghai, China; ^3^College of Chemistry, Chemical Engineering and Biotechnology, Donghua UniversityShanghai, China; ^4^Changhai Hospital of Second Military Medical UniversityShanghai, China; ^5^School of Pharmacy, Shanghai Jiao Tong UniversityShanghai, China

**Keywords:** peripheral nerve injury, nanofibers, conductive polymer, electric stimulation, nerve regeneration

## Abstract

Electrospinning and electric stimulation (ES) are both promising methods to support neuron adhesion and guide extension of neurons for nerve regeneration. Concurrently, all studies focus on either electrospinning for conduits material or ES *in vitro* study to accelerate nerve regeneration; few work on the combined use of these two strategies or ES *in vivo* study. Therefore, this study aimed to investigate the abilities of direct current ES through electrospinning conductive polymer composites composed of polypyrrole and Poly (l-lactic acid-co-ε-caprolactone) (PPY/PLCL) in peripheral nerve regeneration. PPY/PLCL composite conduits were synthesized by polymerizing pyrrole coated electrospun PLCL scaffolds. Morphologies and chemical compositions were characterized by scanning electron microscope and attenuated total reflection fourier transform infrared (ATR-FTIR) microscope. Rat pheochromocytoma 12 (PC12) cells and dorsal root ganglia (DRG) cells cultured on PPY/PLCL scaffolds were stimulated with 100 mV/cm for 4 h per day. The median neurite length and cell viability were measured in PC-12 cells. The levels of brain-derived neurotrophic factor (BDNF), glial cell derived neurotrophic factor (GDNF) and neurotrophin-3 (NT-3) were analyzed in DRG cells. In rats, 15 mm gaps of sciatic nerves were bridged using an autograft, non-stimulated PPY/PLCL conduit and PPY/PLCL conduit stimulated with 100 mV potential, respectively. A 100 mV potential direct current ES was applied for 1 h per day at 1, 3, 5 and 7 days post-implantation. The PPY/PLCL conduits with ES showed a similar performance compared with the autograft group, and significantly better than the non-stimulated PPY/PLCL conduit group. These promising results show that the PPY/PLCL conductive conduits’ combined use with ES has great potential for peripheral nerve regeneration.

## Highlights

1. Conductive PPY/PLCL conduits were synthesized by polymerizing pyrrole coated on Poly (l-lactic acid-co-ε-caprolactone).
2. Efficacies of PPY/PLCL combined with ES were assessed in the rat sciatic nerve 15 mm defects model.
3. Electrical current through PPY/PLCL scaffolds showed similar outcomes with autograft.

## Introduction

More than 100,000 peripheral nerve injuries due to trauma and other reasons cannot be repaired by end to end sutures in the United States and Europe (Schlosshauer et al., 2006). Though the existing gold standard procedure for reconstruction of nerve gap injuries is to utilize autologous nerve grafts, this method has many drawbacks including donor nerve shortage, donor site morbidity and size mismatch (Meek and Coert, 2002; Chen et al., 2006). Nerve tissue engineering offers an alternative approach for treating large nerve defects and many experiments have shown that nerve guidance conduits (NGCs) have the potential to repair peripheral nerve defects (Ouyang et al., 2013; Mu et al., 2014; Li et al., 2015; Pateman et al., 2015).

The ideal NGC should not only bridge gaps across severed nerve defects but also stimulate axons sprouting from the proximal end toward the distal stump (Nguyen et al., 2014). Poly (l-lactic acid-co-ε-caprolactone) (PLCL) is a nontoxic, biodegradable and synthetic co-polymer of poly (L-lactic acid) (PLLA) and poly (ε-caprolactone) (PCL; Jin et al., 2009). PLCL has been investigated as a biomaterial for tissue engineering, such as wound healing, formation of cartilage and cardiovascular applications (Jin et al., 2011; He et al., 2013; Sankaran et al., 2014). Moreover, collagen/PLCL nanofibrous scaffolds and silk fibroin/PLCL nanofibrous scaffolds have been proposed to be considered as potential candidates for nerve tissue engineering (Prabhakaran et al., 2009; Zhang et al., 2013). Electrospinning is a versatile and mainstream technique for obtaining engineer conduits with micro to nanoscale topography and high porosity similar to natural extra cellular matrix (Lannutti et al., 2007; Xu et al., 2013). Conduits generated by electrospinning have been proved to induce nerve regeneration (Weightman et al., 2014). Conductive materials have also attracted much attention to be considered as one of the most promising NGC biomaterials because of their properties of biocompatibility, conductivity and suitable hydrophobicity for cell adhesion (Ravichandran et al., 2010). Polypyrrole (PPY), with the features of good electrical conductivity, biocompatibility and easy to synthesize, is the most widely used scaffold material for nerve tissue engineering research (George et al., 2009; Prabhakaran et al., 2011). It has been reported that polycaprolactone fumarate and PPY composite materials can support cell attachment, proliferation and neurite extension using pheochromocytoma 12 (PC12) cells (Runge et al., 2010). In the study of Xu et al. (2014), composite nerve conduit with PPY and poly (D, L-lactic acid) (PDLLA) showed similar results to the gold standard autologous graft for repair of rat sciatic nerve defect. Therefore, PPY is a promising material for nerve regeneration both *in vivo* and *in vitro* studies. What’s more, electric stimulation (ES) can enhance the progress of nerve regeneration and accelerate axon outgrowth in many *in vitro* studies (Kerns et al., 1991; Gordon et al., 2008; Prabhakaran et al., 2011). Different ES paradigms have been investigated for nerve regeneration, such as pulsed electric fields, direct current and alternating current stimulation (Park et al., 2009; Su and Shih, 2015; Yamaguchi et al., 2016). However, few studies have focused on electrospinning conductive nerve guidance conduit (CNGC) and the combined use with ES for nerve regeneration *in vivo*.

In this study, conductive PPY coated PLCL (PPY/PLCL) nanofibrous conduits were prepared by polymerizing pyrrole coated on electrospun PLCL scaffolds. The biocompatibility and the neuronal differentiation ability of the conductive material film combined with ES were analyzed using rat PC12 cells, which is an extensively used model to study neuronal differentiation (Ravni et al., 2006). We also examined the levels of brain-derived neurotrophic factor (BDNF), glial cell derived neurotrophic factor (GDNF) and neurotrophin-3 (NT-3) in dorsal root ganglia (DRG) cells in different condition. The efficiency of PPY/PLCL conduits combined with direct current ES for nerve regeneration was evaluated using a 15 mm sciatic nerve defects model in Sprague-Dawley rats *in vivo*.

## Materials and Methods

### Fabrication of PLCL Nanofibrous Tubular Scaffolds

The PLCL nanofibrous tubular scaffolds were fabricated by electrospinning with teflon mold collector. At first, PLCL (Mw = 300 kDa, LA:CL = 50:50; Gunze Limited, Japan) was dissolved in 1,1,1,3,3,3-Hexafluoro-2-propanol (HFIP; Alfa Aesar Company, Haverhill, MA, USA) at a concentration of 8% (w/v) at room temperature for 2–4 h with sufficient stirring. Then, the electrospinning solution was fed into a 10 ml plastic syringe and the syringe was loaded in a syringe pump (789100C, Cole-Parmer Instruments, Vernon Hills, IL, USA) with the feeding rate of 1 ml/h. A voltage of 12 kV between the solution and the receiving apparatus was generated by a high-voltage power supply (BGG6-358, BMEI Co. Ltd., China). Fibers were collected with a rotating rate of 50 rpm. The prepared PLCL nanofibrous tubular scaffolds (diameter = 2 mm, length = 5 cm) were placed in vacuum over night to remove the residual solvent prior to use.

### Formation of Polypyrrole Coated PLCL Nanofibrous Scaffolds

The prepared PLCL nanofibrous tubular scaffold was immersed in 40 ml aqueous solution of 10 mM pyrrole (Sigma-Aldrich, St. Louis, MO, USA) and 10 mM P-toluene sulfonate with stirring at 0°C for 1 h. Then, deposition of PPY coating was initiated with the addition of 200 μl ferric chloride at 0°C. PPY coated PLCL nanofibrous scaffolds were taken out 6 h (PPY/PLCL-1) and 12 h (PPY/PLCL-2) later. The PPY/PLCL nanofibrous scaffolds were washed with deionized water and ethanol at least three times, respectively. Finally, PPY/PLCL scaffolds were dried in vacuum for subsequent use.

### Characterization of PPY/PLCL Conduits

The morphology of the fabricated nanofibers was observed using a JSM-5610LV scanning electron microscope (JEOL, Japan) with an accelerating voltage of 10 kV. Prior to observation, the specimens were sputter-coated with gold. Diameter of the nanofibers was calculated by ImageJ software (Bethesda, MD, USA). The attenuated total reflection fourier transform infrared (ATR-FTIR) spectroscopy was used to identify the chemical components on nanofibers using a Nicolet Nexus FTIR Spectrometer (Nexus, Thermo Scientific, Waltham, MA, USA). The conductivity of PPY/PLCL-1 and PPY/PLCL-2 was measured by the 4-point probe method using the Hall Effect testing system (HL5500PC, Accent Optical, UK).

### Assessment of PPY/PLCL Scaffolds *in Vitro*

PC12 and DRG cell lines were purchased from the cell bank of the Chinese Academy of Sciences (Shanghai, China). PC12 cells were cultured in F-12K medium (ATCC, Manassas, VA, USA) supplemented with 15% heat-inactivated horse serum (Gibcol, Life Technologies, Carlsbad, CA, USA), 2.5% fetal bovine serum (Gibcol, USA) and 1% Penicillin/Streptomycin solution (Gibcol, USA). PC12 cells were used to determine the cytotoxicity of ES and CNGC. DRG cells were maintained in RPMI 1640 media supplemented with 10% FBS, 1% PSA and 50 ng/ml NGF. Cells were incubated at 37°C in a humid incubator with 5% CO_2_. Cells were seeded on the membranes at a density of 1 × 10^4^ cell/cm^2^ and cultured for 24 h in our special culture dish, where PPY/PLCL film was placed in the dish bottom and two platinum wire attached to the film as the anode and cathode (Figure 5F▪). ES was conducted by a voltage of 100 mV/cm through the conductive polymers for 4 h per day. The tissue culture plates (TCP) were used as control. After 1, 3, 5 and 7 days, the neurite length and cell viability of PC12 cells seeded on TCP, the conductive PPY/PLCL membrane without ES and with ES were analyzed. Briefly, PC12 cells grown on the PPY/PLCL film and the TCP were fixed with 4% paraformaldehyde solution at 4°C for 1 h. Then all samples were washed twice in PBS solution for 20 min, dehydrated in ethanol with sequentially increasing concentrations (50–100%) and dried in a CO_2_ critical point dryer to remove the ethanol fully. Then a slight thick gold layer was coated on the sample and SEM (JSM-5600, Japan) was used to observe and analyze the sample. At least 500 PC12 cells were examined for each film and condition and all procedures were performed by following a previous study (Leach et al., 2007). The cell viability in each group was measured by cell counting Kit-8 (CCK-8) regent. Appropriate amount CCK-8 regent was added into each dish and cultured for 2 h and measured at 450 nm and 600 nm using multifunctional microplate reader (SpectraMax M3 Multi-Mode Microplate Reader). TCP was used as control. Six samples (*n* = 6) for each film and condition were studied.

The levels of GDNF, BDNF and NT-3 in DRG cells were examined by ELISA measurement, Western blot assay and real time RT-PCR analysis. ELISA measurement was used to evaluate neurotrophic protein expression in different groups in the supernatant according to the manufacturer’s instructions (R&D Systems, Minneapolis, MN, USA). Western blot assay was also used to examine the GDNF, BDNF and NT-3 expression. All three groups of cells were washed in 0.1 M PBS on ice, and lysed in RIPA buffer including 0.005 M Tris, 0.001 M EDTA, 100 μg/ml PMSF, 1 mM activated sodium orthovanadate 24 h after ES. Lysed cells were collected by centrifugation at 1500 rpm for 15 min at 4°C to obtain total protein. The protein concentrations were determined using the bicinchoninic acid (BCA) assay kit (Beyotime), following the manufacturer’s protocol using GAPDH as the control.

The antibodies used in this procedure were as follows: anti-rabbit GDNF (1:1000, Abcam, Cambridge, MA, USA), anti-rabbit polyclonal BDNF (1:1000, Abcam), anti-rabbit NT-3 (1:1000, Abcam) as primary antibodies and horseradish peroxidase (HRP)-labeled secondary antibody (1:3000, Santa Cruz Biotech, Santa Cruz, CA, USA). Western blot assay was performed at least twice for each sample and quantified using ImageJ software. Total mRNAs were extracted from the cells using RNeasy^®^Mini Kit (Qiagen, Valencia, CA, USA) and reverse transcribed into cDNA using High-Capacity cDNA Reverse Transcription Kit (Applied Biosystems). The primer sequences of GDNF, BDNF and NT-3 were GDNF (forward: CCAGAGAATTCCAGAGGGAAA GGT, reverse: TCAGTTCCTCCTTGGTTTCGTAGC), BDNF (forward: ATCCCATGGGTTACACGAAGGAAG, reverse: GTAAGGGCCCGAACATACGATTG) and NT-3 (forward: GATCCAGGCGGATATCTTGA, reverse: AGCGTCTCTGTTGC CGTAGT). The expression of GDNF, BDNF and NT-3 was analyzed by real-time RT-PCR normalized to GAPDH followed in previous study (Kingham et al., 2013).

### Animal and Surgical Procedures

Thirty Sprague Dawley rats (male, weighing 200–250 g) were housed under standard laboratory conditions. These animals were randomly divided into three groups (*n* = 10): PPY/PLCL CNGC group (group I, PPY/PLCL), PPY/PLCL CNGC combined with ES group (group II, PPY/PLCL + ES) and autograft group (group III). The animals were anesthetized by intraperitoneal injection of 50 mg/kg pentobarbital sodium and a 3-cm incision was made on the right thigh to expose the sciatic nerve under sterile conditions. A 15 mm segment of sciatic nerve was resected and removed at the mid-thigh level, causing a defect gap between two nerve stumps. Subsequently, PPY/PLCL conduit was sutured to bridge the nerve defects of animals in group I and group II. For group III, the resected nerve was reversed 180° and implanted across the defect. The 8–0 nylon sutures were used to suture the proximal and distal nerve stumps. In all groups a nickel-titanium alloy wire was placed into the proximal and distal segments with a 1/4 circle electrode and buried in neck through the subcutaneous tunnel (Figure 5F°). Then, the muscle and skin incision were closed with 4–0 silk sutures. ES was applied by a 100 mV potential through the wires for 1 h on 1, 3, 5 and 7 days post-implantation under anesthesia for group II. Group I and III underwent anesthesia at the same time without ES. All groups underwent a second surgical procedure to remove the wire and electrode when the ES program was finished at day 7. All rats after surgery were housed under standardized laboratory conditions and monitored to observe changes in ordinary conditions and activities. Animal care and use were in accordance with the guidelines established by the Animal Ethics Committee for Shanghai JiaoTong University.

### Electrophysiological and Functional Evaluation

Sciatic function index (SFI) was calculated to assess the recovery of nerve function by the walking track analysis at 4 and 8 weeks post-implantation (de Medinaceli et al., 1982). Meanwhile, digital MYTO electromyograph machine (Esaote, Genoa, Italy) was used to evaluate the nerve conduction velocity (NCV) and distal compound motor action potential (DCMAP) at 4 and 8 weeks post-implantation. Animals were anesthetized with intraperitoneal pentobarbital sodium (same doses used for the surgical procedure) and the NGC in groups I and II were removed to expose the sciatic nerve. Bipolar stimulating electrodes were applied to the sciatic nerve trunk at its proximal portion and a monopolar recording electrode in the gastrocnemius muscle to record the NCV and DCMAP. Moreover, triceps surae muscles of the left (contralateral unoperated) and right (operated) sides from the anesthesia-killed rats were carefully resected and weighed at 4 and 8 weeks post-implantation. The recovery rate of triceps surae muscles was presented as a ratio of right side muscle weight compared to that of the left side (Yu et al., 2012).

### Histological Analysis and Immunofluorescence Staining

Immediately after electrophysiological assessment, the right regenerated nerve (1 cm) was rapidly removed (*n* = 5 for each group) at 4 and 8 weeks post-implantation. Transverse sections (5 μm thick) in the middle segment of the regenerated nerve were prepared for histological analysis by 1% toluidine blue staining and transmission electron microscopy (TEM). Axonal regeneration was investigated by Image-Pro Plus 6.0 (Media Cybernetics, Rockville, MD, USA) to calculate the diameter of axons and nerve fibers, count the number of nerve fibers and obtain the thickness of the myelin sheaths in each section of the nerve. The procedure has been introduced in our study previously (Wang et al., 2011).

Triple immunofluorescence staining was used to analyze the regenerated nerves at the middle of the conduit. Specimens of nerves were fixed with 2.5 v/v % glutaraldehyde in 0.1 M PBS (PH 7.4) at 4°C for 48 h. Then the nerve segments were fixed with 1% osmium tetroxide fixed, dehydrated and embedded in Epon 812 (Electron Microscopy Sciences, Hatifield, PA, USA) resin. The cross sections were cut at 5 μm thick (Leica EM UC6 ultramicrotome) and mounted on gelatin pre-coated slides. Then the sections were incubated with primary antibody (S100, 1:200, rabbit, Abcam, UK; neurofilament 160 (NF160), 1:1000, mouse, Abcam, UK) overnight at 4°C followed by two washes with 0.03% Triton-X 100. Then samples were incubated with Alexa Fluoro 555 red goat anti-rabbit IgG secondary antibody and FITC green goat anti-mouse IgG secondary antibody (1:200, Beyotime, USA) for 1 h at room temperature. Cell nuclei were counter stained with 0.1% of 4,6-diamidino-2-phenylindole (DAPI) for 5 min (1:100). Finally, the stained sections were viewed on a fluorescence microscope (Olympus, Tokyo, Japan). The percentage of positive area was analyzed by Image-Pro Plus software for quantitative results.

### Statistical Analysis

All data were expressed as mean ± standard deviation (SD) and all tests were repeated more than three times in each group. For comparisons among three groups, statistical analysis was performed using one-way analysis of variance (ANOVA) followed by Tukey’s *post hoc* test using SPSS 11.0 software for Windows student version. *P* < 0.05 was considered statistically significant.

## Results

### Morphologies of the Nanofibrous Scaffolds

**Figure 1:**
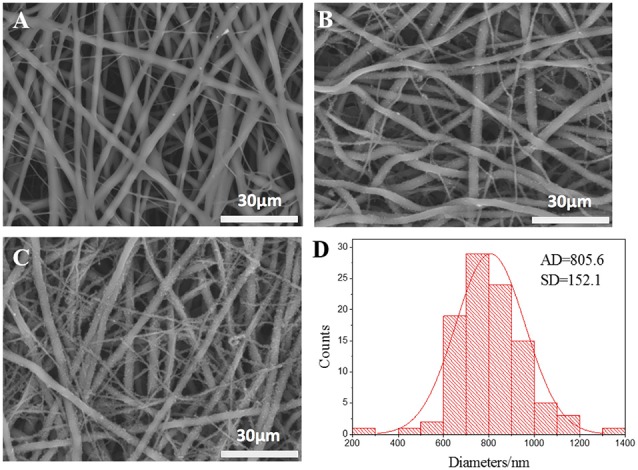
**The scanning electron microscope images of electrospun nanofibers. (A)** Poly (l-lactic acid-co-ε-caprolactone) (PLCL) nanofibers; **(B)** Polypyrrole (PPY)/PLCL-1 nanofibers; **(C)** PPY/PLCL-2 nanofibers; **(D)** diameter distribution of PLCL/PPY-2 nanofibers (AD: average diameter).

The micrographs of nanofibrous tubular scaffolds of PLCL, PPY/PLCL-1 and PPY/PLCL-2 showed relatively uniform fiber morphologies without beads (Figure 1). PLCL nanofibers were smooth because of the main chain of P(LLA-CL) molecules as single C–C bond and the molecular chain segments moved in a flexible way (Figure 1A). However, the PPY/PLCL-1 (Figure 1B) and PPY/PLCL-2 (Figure 1C) nanofibers had rough surface since there were many PPY nanoparticles coated on PLCL nanofibers. Moreover, the surface of PPY/PLCL-2 nanofibers was much rougher than PPY/PLCL-1 due to the oxidative polymerization time of PPY/PLCL-2 conduits which was much higher than that of PPY/PLCL-1 conduits. The PPY/PLCL-2 consisted of many nanoscale fibers with a mean diameter of 805.6 ± 152.1 nm from randomly selected six visual fields (Figure 1D).

### Characterization of PPY/PLCL Conduits

**Figure 2:**
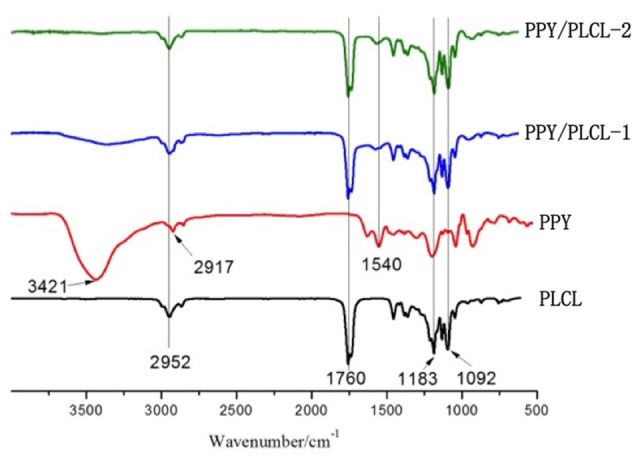
**The attenuated total reflection fourier transform infrared (ATR-FTIR) spectroscopy of PLCL nanofibers, PPY, PPY/PLCL-1 nanofibers (6 h) and PPY/PLCL-2 nanofibers (12 h)**.

The FTIR spectra (4000–500 cm^−1^) of raw PPY, PLCL nanofibers, PPY/PLCL-1 and PPY/PLCL-2 nanofibers are shown in the Figure 2. There is an absorption peak in PLCL, PPY/PLCL-1 and PPY/PLCL-2 nanofibers spectra at 2952 cm^−1^ corresponding to -CH_3_ or -CH_2_ stretching vibration in PLCL molecular skeleton. Three representative adsorption peaks at 1760, 1183 and 1092 cm^−1^, respectively correspond to—COOR and C–O stretching vibration in PLCL, PPY/PLCL-1 and PPY/PLCL-2 nanofibers. For raw PPY, one characteristic absorption peak at 1540 cm^−1^ corresponding to the C = C stretching vibration can also be found in PPY/PLCL-1 and PLCL/PPY-2 nanofibers. There were two absorption peaks at 3421 cm^−1^ and 2917 cm^−1^ in raw PPY, which correspond to N-H and -CH_2_. However, the absorption peak at 2917 cm^−1^ of PPY was overlapped with PLCL and the absorption peak at 3421 cm^−1^ was not obvious in PLCL/PPY-1 and PPY/PLCL-2 nanofibers because the N-H of PPY reacted with PLCL and coated on the surface of PLCL nanofibers. Together, these results indicated the existence of PPY in PPY/PLCL-1 and PLCL/PPY-2 nanofibers. The conductivity of PPY/PLCL-2 was 6.72 × 10^−5^ S/cm, higher than that of PPY/PLCL-1 (2.41 × 10^−5^ S/cm). PPY/PLCL-2 nanofibers was chosen for further *in vivo* and *in vitro* study because of its rougher surface and better conductivity.

### *In Vitro* Assessment of PPY/PLCL Scaffolds

**Figure 3:**
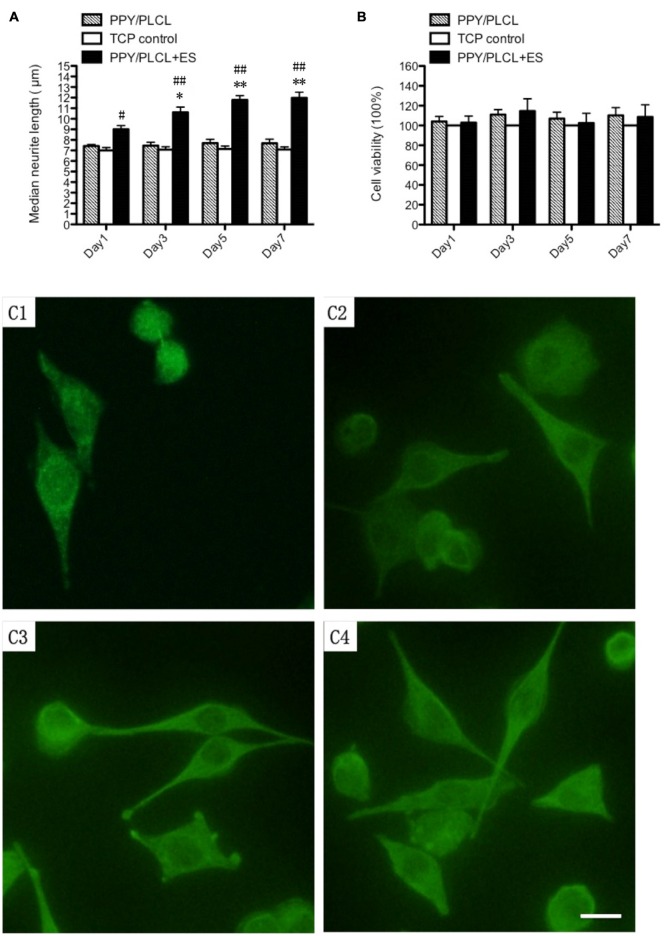
***In vitro* study of pheochromocytoma (PC12) cells. (A)** Median neurite length. **(B)** Cell viability by cell counting Kit-8 (CCK-8). All data from the tissue culture plates (TCP; control group), conductive PPY/PLCL film without electric stimulation (ES; PPY/PLCL group) and with ES (PPY/PLCL + ES group) on Day 1, 3, 5 and 7 (*n* = 6, ^#^*p* < 0.05, ^##^*p* < 0.01 the PPY/PLCL + ES group vs. TCP control group; *p < 0.05, **p < 0.01 the PPY/PLCL + ES group vs. PPY/PLCL group). **(C)** Fluorescence images of PC12 cells cultured on PPY/PLCL film with ES (E1: Day1, E2: day3, E3: Day 5, E4: Day 7; scale bar: 40 μm).

Compared with the conductive PPY/PLCL film without ES, the PPY/PLCL film combined with ES significantly elevated the median neurite length on days 1, 3, 5 and 7 (*p* < 0.05; Figure 3A). Neurite outgrowth was significantly increased by ES and the effect was more obvious over time (Figures 3C1–C4). Meanwhile, the quantity of PC12 cells was gradually increased along with the culture time in all three groups and the cell viability of PC12 cells on PPY/PLCL film combined with ES showed no significant difference compared with the conductive PPY/PLCL film without ES and the TCP control group (*p* > 0.05; Figure 3B).

**Figure 4:**
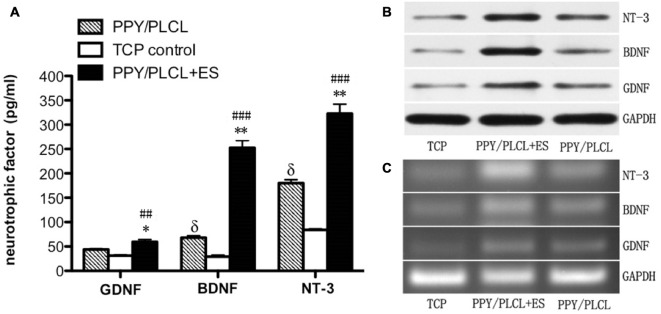
**Glial cell derived neurotrophic factor (GDNF), brain-derived neurotrophic factor (BDNF) and neurotrophin-3 (NT-3) expression in dorsal root ganglia (DRG) cells 24 h after ES. (A)** ELISA measurement. **(B)** Protein levels by Western blot assay. **(C)** Relative mRNA expression by RT-PCR (*n* = 6, ^##^*p* < 0.01, ^###^*p* < 0.001 the PPY/PLCL + ES group vs. TCP control group; *p < 0.05, **p < 0.01 the PPY/PLCL + ES group vs. PPY/PLCL group; ^δ^*p* < 0.05, the PPY/PLCL group vs. TCP control group).

The concentration of GDNF, BDNF and NT-3 in the DRG cell supernatant in all three groups was detected by ELISA measurement 24 h after ES. As shown in Figure 4A, the concentration of BDNF secretion was about 260 pg/ml in the PPY/PLCL + ES group, which was approximately increased 4-fold compared with the PPY/PLCL group (*p* < 0.01, *n* = 6). There was a significant difference in the concentration of BDNF secretion between the conductive PPY/PLCL film group and the TCP control group (*p* < 0.05, *n* = 6). The concentration tendency of GDNF and NT-3 in three groups was in accordance with that of BDNF. Similar results were found by Western blot assay (Figure 4B). The messenger RNA (mRNA) expression of GDNF, BDNF and NT-3 was analyzed by RT-PCR and the results were shown in Figure 4C. The expression of GDNF mRNA in DRG cells on the conductive PPY/PLCL film (with and without ES) was significantly upregulated compared with the TCP control group (*p* < 0.05, *n* = 6). Meanwhile, ES was applied on the conductive PPY/PLCL film and the GDNF mRNA expression was significantly higher than that without ES group (*p* < 0.01, *n* = 6). The results of RT-PCR were correlated with ELISA measurement and Western blot assay.

### Animal Operation Procedure

**Figure 5:**
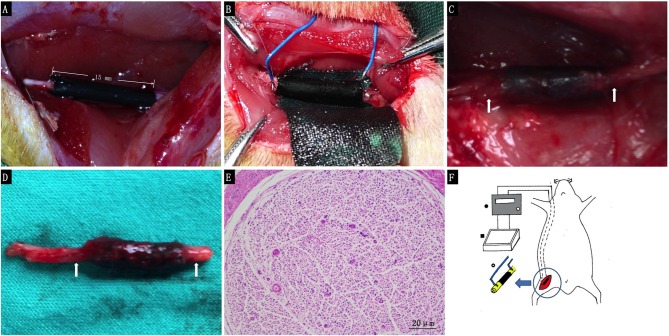
**The animal operation procedure. (A)** Immediately after 15 mm conduit implantation. **(B)** 1/4 circle electrode implantation. **(C)** Eight weeks post-implantation. **(D)** Harvested regenerated nerve at 8 weeks post-implantation. **(E)** Histological section of the electrode contact site stained with hematoxylin and eosin 8 weeks post-implantation (White arrows: the electrode contact site). **(F)** Schematic illustration for animal procedure: a 15 mm sciatic nerve defect was bridged by PPY/PLCL conduits (right thigh, black), nickel-titanium alloy wire was placed into the proximal and distal segments with a 1/4 circle electrode and buried behind the neck through the sub cutaneous tunnel (• Electronic Stimulator ▪ special made cell culture dish **°** tact site of the electrodes to the conduit).

For the *in vivo* study, we used a 15 mm defect in the sciatic nerve of adult SD rats as a model. PPY/PLCL conductive conduits were used to repair the defects in group I and II. In group II, the nickel-titanium alloy wire was placed into the proximal and distal segments with a 1/4 circle electrode and buried in neck through the subcutaneous tunnel. All 30 animals in this experiment showed no severe complications during and post operation. Neither inflammatory reactions nor complications typically associated with the operation were observed, indicating good tissue response of the synthetic conduit. At 4 and 8 weeks post-implantation, five animals of each group were harvested for further study. At 8 weeks post-implantation, the conduits showed obvious degradation and no severe compression by the electrode was observed (Figures 5A–F).

### Electrophysiological and Functional Evaluation

**Figure 6:**
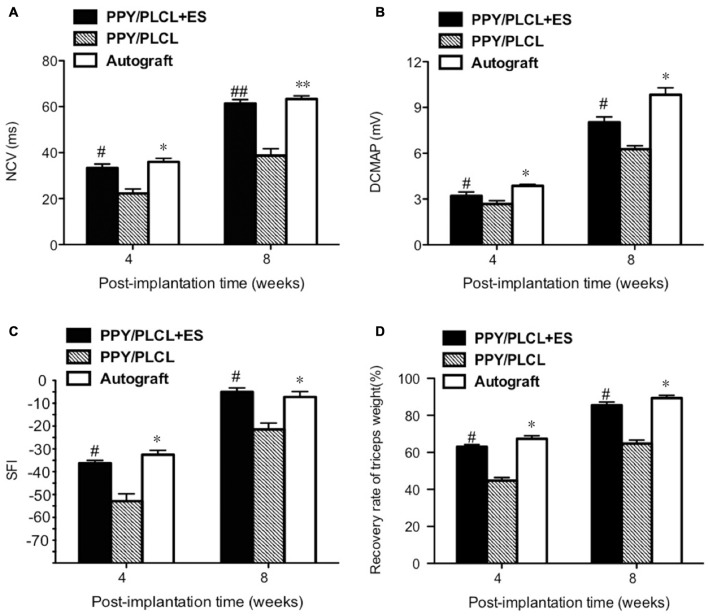
**Electrophysiological and functional evaluation 4 and 8 weeks post-implantation. (A)** Nerve conduction velocity (NCV). **(B)** Distal compound motor action potential (DCMAP). **(C)** Sciatic function index (SFI). **(D)** Recovery rate of triceps weight (*n* = 5, ^#^*p* < 0.05, ^##^*p* < 0.01 the PPY/PLCL + ES group vs. PPY/PLCL group; *p < 0.05, **p < 0.01 the autograft group vs. PPY/PLCL group).

The NCV of the PPY/PLCL group was significantly slower than that of the PPY/PLCL + ES group (*n* = 5, *p* < 0.05). Moreover, there was no significant difference between the PPY/PLCL + ES group and the autograft group at 4 and 8 weeks post-implantation (Figure 6A). At 8 weeks post-implantation, the NCV for the PPY/PLCL group, PPY/PLCL + ES group and the autograft group was 41.23 ± 1.54 m/s, 61.34 ± 4.21 m/s and 63.32 ± 2.54 m/s, respectively. At 4 weeks post-implantation, the DCMAP of the PPY/PLCL + ES group (3.21 ± 0.14 mV) and the autograft group (3.87 ± 0.23 mV) were significantly higher than that of the PPY/PLCL group (2.67 ± 0.27 mV; *n* = 5, *p* < 0.05). The DCMAP of the PPY/PLCL group (6.27 ± 0.14 mV) was lower than those of the PPY/PLCL + ES group (8.07 ± 0.24 mV) and the autograft group (9.34 ± 0.12 mV) at 8 weeks post-implantation (*n* = 6, *p* < 0.05; Figure 6B).

At 4 weeks post-implantation, the SFI of the PPY/PLCL group (−52.5 ± 2.1) was significantly lower than that of the PPY/PLCL + ES group (−44.7 ± 2.7) and the autograft group (−42.1 ± 1.8; *n* = 5, *p* < 0.05), while there was no significant difference between the PPY/PLCL + ES group and the autograft group (*n* = 5, *p* > 0.05; Figure 6C). At 8 weeks post-implantation, the SFI of the PPY/PLCL group, the PPY/PLCL + ES group and the autograft group came to −34.1 ± 2.1, −23.5 ± 1.2 and −21.4 ± 1.1, which showed the similar changes as 4 weeks post-implantation. The recovery rates of triceps surae muscles of PPY/PLCL + ES group and the autograft group were significantly higher than that of the PPY/PLCL group (*n* = 5, *p* < 0.05; Figure 6D). There was no significant difference between the PPY/PLCL + ES group and the autograft group (*n* = 5, *p* > 0.05).

### Histological and Immunofluorescence Analysis

**Figure 7:**
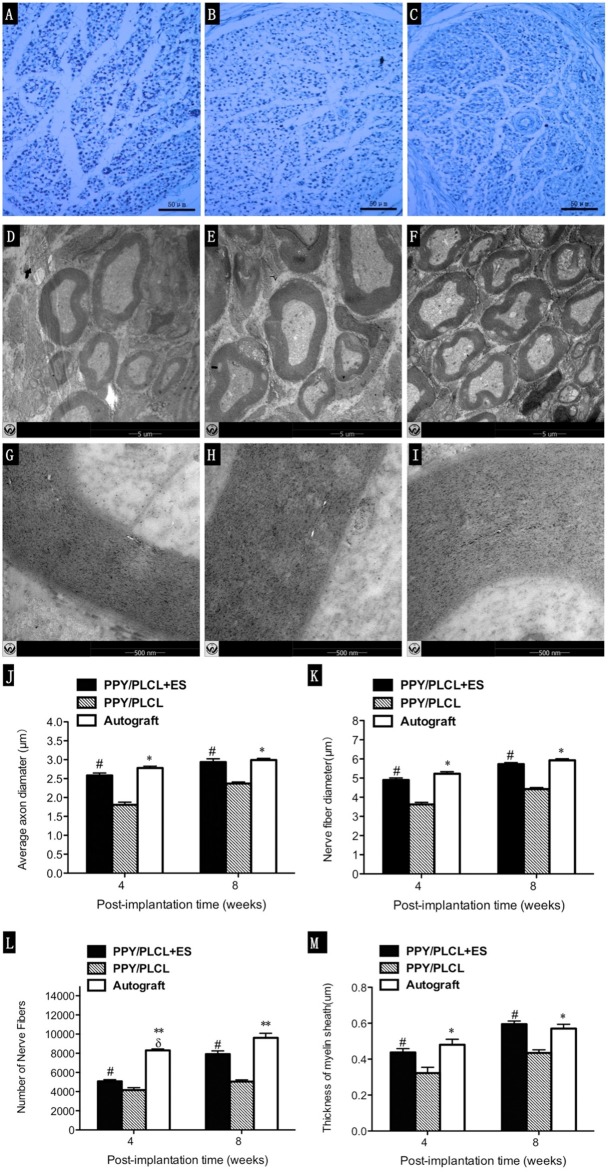
**Histology images stained with toluidine blue and transmission electron microscopy (TEM) micrographs.** Images represent cross sections of regenerated nerves taken from different conduits in rats at 8 weeks post-implantation. **(A,D,G)** From the PPY/PLCL group, **(B,E,H)** from the PPY/PLCL + ES group and **(C,F,I)** from the autograft group. Histology scale bar: 50 μm **(A–C)**. TEM scale bar: 5 μm **(D–F)**, 500 nm **(G–I)**. **(J)** Average axon diameter. **(K)** Nerve fiber diameter. **(L)** Number of nerve fibers. **(M)** Thickness of myelin sheath (*n* = 5, ^#^*p* < 0.05 the PPY/PLCL + ES group vs. PPY/PLCL group; *p < 0.05, **p < 0.01 the autograft group vs. PPY/PLCL group; ^δ^*p* < 0.05, the autograft group vs. PPY/PLCL + ES group).

The total myelinated fiber counts, the myelinated fiber diameter and the average axon diameter of the PPY/PLCL + ES group were significantly larger compared with the PPY/PLCL group (*p* < 0.05, Figures 7J–M). However, there was no significant difference between the PPY/PLCL + ES group and the autograft group (*p* > 0.05). The results of toluidine blue staining showed that there were more nerve fibers in the PPY/PLCL + ES group than that of the PPY/PLCL group at 8 weeks post-implantation (Figures 7A–C). TEM showed that the myelin sheath thickness was significantly greater in the PPY/PLCL + ES group compared with the PPY/PLCL group at 4 weeks post-implantation (0.51 ± 0.08 μm vs. 0.36 ± 0.11 μm; *p* < 0.05). Meanwhile, no significant difference was observed in the thickness of the myelin sheath between the PPY/PLCL + ES group and the autograft group (*p* > 0.05, Figures 7D–I).

**Figure 8:**
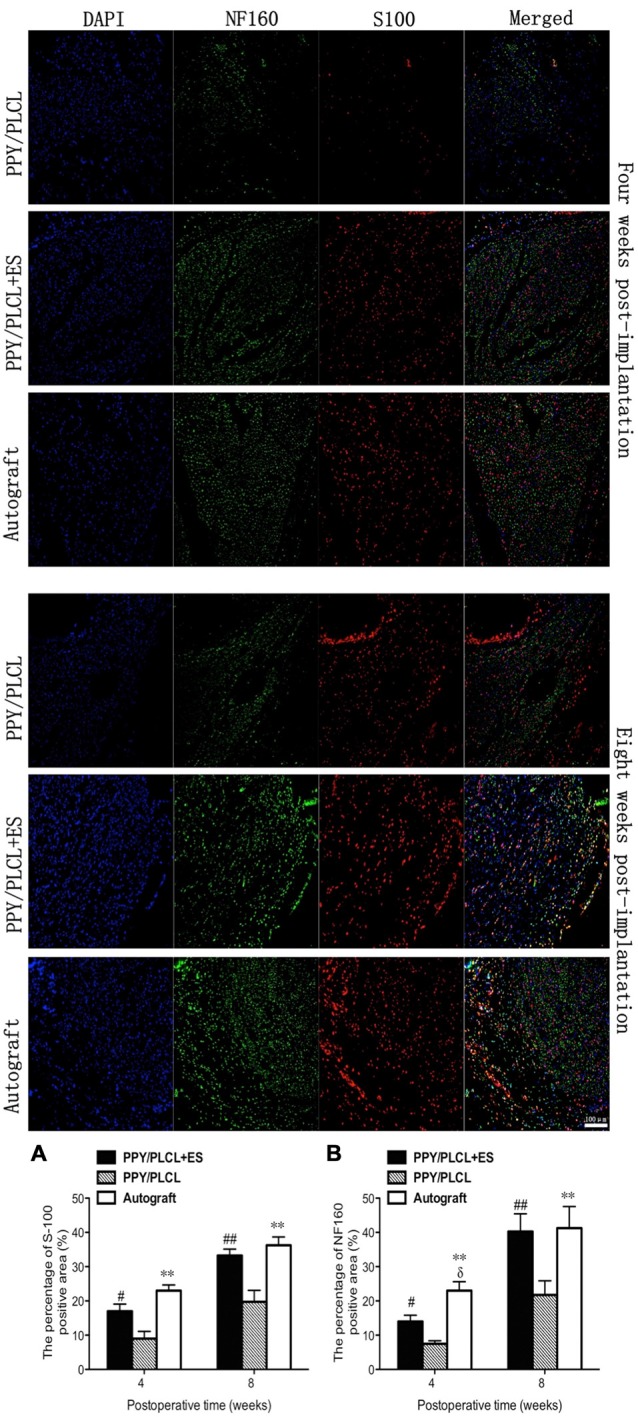
**Immunofluorescence analysis of neurofilament 160 (NF160) and S100 in the middle of the sciatic nerve at 4 and 8 weeks post-implantation.** The percentages of S100 and NF160 positive area for each group are shown in **(A,B)** (*n* = 5, ^#^*p* < 0.05, ^##^*p* < 0.01 the PPY/PLCL + ES group vs. PPY/PLCL group; *p < 0.05, **p < 0.01 the autograft group vs. PPY/PLCL group; ^δ^*p* < 0.05, the autograft group vs. PPY/PLCL + ES group).

NF160-positive axons and S100-positive Schwann cells (SCs) were found in the middle of the conduit in all these three groups at 4 and 8 weeks post-operation (Figure 8). Both the number of NF160-positive axons and S100-positive SCs were obviously larger at 8 weeks post-operation than those at 4 weeks post-operation. Interestingly, more host-derived SC entered the middle of the conduit when ES was applied where more NF160 positive axons were observed. The percentages of positive area of PPY/PLCL + ES group and the autograft group were significantly higher at 8 weeks after surgery, comparing with the PPY/PLCL group (*p* < 0.01). There was no significant difference in percentages of positive area between the PPY/PLCL + ES group and the autograft group (*p* > 0.05; Figures 8A,B).

## Discussion

It would be beneficial for peripheral nerve regeneration to develop NGCs that match the effectiveness of autologous nerve graft since autologous nerve graft is associated with a variety of clinical complications (Nectow et al., 2012). A superior NGC is crucial for direction of cells towards a targeted functional outcome by providing appropriate chemical, morphological and structural cues (Wang et al., 2011). In this study, we explored the application of ES through a conductive PPY/PLCL composite conduit synthesized by polymerization of PPY coated electrospun PLCL nanofibrous scaffolds for peripheral nerve regeneration.

Electrospinning using synthetic polymers is an effective fabrication technique to make nanofibrous scaffolds (Jiang et al., 2010). PPY/PLCL scaffolds consisted of relatively uniform nanoscale fibers of 805.6 ± 152.1 nm in diameter which were produced by electrospinning in our study. *In vitro* study, no significant architecture change of the film in the 100 mV/cm electric field was observed at day 7, indicating that the PPY/PLCL conductive fibers are electrically stable, at least for 1 week. PPY/PLCL fibers were biodegradable over time and the complete conduit structure was undetectable at 8 weeks post-implantation. Importantly, the rough surface of PPY/PLCL scaffolds due to PPY particles coated over PLCL film makes it proper for cell adhesion and proliferation (Ibrahim et al., 2016). Conductive materials along with ES can promote PC12 cell proliferation, migration and neurite extension (Kang et al., 2011; Pires et al., 2015; Su and Shih, 2015). The greatest disadvantage of conductive materials is its marginal biodegradation. So we chose PPY/PLCL-2 for further study because of its satisfactory conductivity at a relative low PPY concentration. *In vitro* culture, electrical stimulation of PC12 cells cultured on PPY/PLCL film with a voltage of 100 mV/cm for 4 h resulted in significantly extended neurite outgrowth and increased proliferation of cells compared to the cells grown on non-stimulated PPY/PLCL film, demonstrating its differentiation promotion effect on PC12 cells. Meanwhile, both the conductive PPY/PLCL film and the application of ES didn’t affect the cell viability, showing their property of non-toxic and good biocompatibility on PC12 cells. Both conductive materials and ES play positive roles in promoting nerve regeneration (Xu et al., 2014). In DRG cells, PPY/PLCL film with ES significantly promoted the expression of GDNF, BDNF and NT-3, which was important for nerve regeneration. Our *in vitro* study results demonstrated that ES was good for nerve regeneration and the increased NT expression might make a contribution. Thus, PPY/PLCL scaffolds with electrical stimulation were nontoxic and biocompatible and promoted neurotrophic factor expression, suggesting its promising potential for peripheral nerve regeneration.

It has been reported that applying ES after surgical repair can accelerate axon regeneration and muscle reinnervation (Gordon et al., 2008). Sciatic nerve model with adequate length and space at the mid-thigh is the most commonly used nerve regeneration studies (Rodríguez et al., 2004). In our animal experiment, ES through conductive conduits was applied in the *in vivo* study for the first time and got a similar outcome to the autograft group. Sciatic nerve model was established to test the efficacy of ES through PPY/PLCL conduits in bridging a 15 mm sciatic nerve defect gap. In our study, NCV of PPY/PLCL with ES group and the autograft group was respectively 61.34 ± 4.21 m/s and 63.32 ± 2.54 m/s at 8 weeks post-implantation. Nerve conduction velocities in healthy subjects can range from 50 m/s to 70 m/s (Kasius et al., 2014). DCMAP is a commonly used parameter reflecting the number of regenerated motor nerve fibers and the extent of muscle reinnervation (Wolthers et al., 2005). No significant difference was observed in the NCV and DCMAP between the PPY/PLCL + ES group and the autograft group. Triceps surae muscle weight ratio can be used to assess the efficacy of reinnervation since balance between protein synthesis and degradation is destroyed by nerve injury leading to muscle weight loss (Vleggeert-Lankamp, 2007). Besides, there was no statistical difference in the SFI and recovery rate of triceps surae muscles between the PPY/PLCL with ES group and the autograft group. Therefore, these data suggested that PPY/PLCL + ES group achieved a rapid functional recovery similar to autograft group. It has been reported that NCV and mean fiber diameter were the most reliable indices of functional recovery during sciatic nerve regeneration (Ikeda and Oka, 2012). Moreover, NCVs critically depend on the diameter of the axons, myelin sheath thickness and internode length (Simpson et al., 2013). In our histological examination, regenerated nerves from PPY/PLCL + ES and autograft showed comparable total myelinated fiber counts, myelinated fiber diameter, average axon diameter and myelin sheath thickness. SCs can produce various neurotrophic factors which are very important for axonal regeneration and neuronal repair after peripheral nerve injury (Martini et al., 2008). Electric field has a large effect on SCs migration and neurite outgrowth (Koppes et al., 2011; Forciniti et al., 2014). Triple immunofluorescence staining analyses showed that there was no significant difference in the number of NF160-positive axons and S100-positive SCs between the PPY/PLCL + ES group and the autograft group. ES attracted more SCs into the conduit and a better regeneration outcome was achieved. In short, the application of ES had a significant positive effect on the axon regeneration and myelination.

In conclusion, we successfully synthesized PPY/PLCL composite conduits by polymerizing pyrrole on PLCL scaffolds fabricated by electrospinning. Our *in vitro* and *in vivo* studies suggested that passing an electrical current through PPY/PLCL conduits is promising for stimulating and guiding peripheral nerve functional regeneration, representing similar efficiencies with autologous graft. Thus, PPY/PLCL NGCs with ES might have potential applications in nerve regeneration.

## Conclusion

In this study, electrospinning and ES were used for nerve regeneration both *in vivo* and *in vitro* studies, got the results of satisfying functional recovery and equivalent morphological recovery to nerve autografts. Our study was a preliminary research on conductive conduits combined with ES for nerve regeneration, while more work about ES mode selection and device improvement should be done. PPY/PLCL conductive conduit’s combined use with ES seems to improve nerve regeneration and functional recovery, which offers a promising approach to repair long-segment nerve defect.

## Author Contributions

JS, BS, SL, WC, YZ, CW, XM, JC, YO, WY and CF conceived and participated in its design, did tests, searched databases, extracted and assessed studies and helped to draft the manuscript. YO, WY and CF participated in the conceptualization and design of the experiment, data extraction and analysis. JS and BS wrote the manuscript. YO, WY and CF revised the manuscript. All authors read and approved the final manuscript.

## Conflict of Interest Statement

The authors declare that the research was conducted in the absence of any commercial or financial relationships that could be construed as a potential conflict of interest. The reviewer NB and handling Editor declared their shared affiliation, and the handling Editor states that the process nevertheless met the standards of a fair and objective review.
